# Supplementary material for: From sequence to enzyme mechanism using multi-label machine learning
Source: BMC Bioinformatics. 2014 May 19;15:150. doi: 10.1186/1471-2105-15-150 (PMC4229970; doi:10.1186/1471-2105-15-150)
Supplement: Additional file 2 — Java code of ml2db. Additional file ml2db_code.tar.gz contains the Java source code to run the multi-label machine learning experiments and save the results to database. The code’s Javadoc is included. [file 1471-2105-15-150-S2.zip › additional file 2/ml2db/ml2db/doc/main/ml2db/mulan/mulanarff/package-use.html]

Uses of Package main.ml2db.mulan.mulanarff


JavaScript is disabled on your browser.


- Overview
- Package
- Class
- Use
- Tree
- Deprecated
- Index
- Help

- Prev
- Next

- Frames
- No Frames

- All Classes

# Uses of Package main.ml2db.mulan.mulanarff

- Packages that use main.ml2db.mulan.mulanarff

  | Package | Description |
  | --- | --- |
  |  |  |
  | --- | --- |
  | main.ml2db.al |  |
  | main.ml2db.al.learning |  |
  | main.ml2db.al.utility |  |
  | main.ml2db.al.utility.confidence |  |
  | main.ml2db.al.utility.globaldistance |  |
  | main.ml2db.al.utility.hybrid |  |
  | main.ml2db.al.utility.neighbours |  |
  | main.ml2db.al.utility.pickers |  |
  | main.ml2db.al.utility.pickers.byfrequency |  |
  | main.ml2db.graphviz |  |
  | main.ml2db.mulan.attributesfiltered |  |
  | main.ml2db.mulan.learn.crosseval |  |
  | main.ml2db.mulan.learn.traintest |  |
  | main.ml2db.mulan.mulanarff |  |
  | main.ml2db.mulan.mulanarff.database |  |
  | main.ml2db.mulan.predict |  |
  | main.ml2db.weka |  |
  | test.ml2db.al |  |
  | test.ml2db.al.utility.confidence |  |
  | test.ml2db.al.utility.globaldistance |  |
  | test.ml2db.mulan.mulanarff |  |
- Classes in main.ml2db.mulan.mulanarff used by main.ml2db.al

  Class and Description

  IdMultiLabelInstances

  A set of identified Mulan Multi Label instances (each instance has a unique
  string identifier).
- Classes in main.ml2db.mulan.mulanarff used by main.ml2db.al.learning

  Class and Description

  IdMultiLabelInstances

  A set of identified Mulan Multi Label instances (each instance has a unique
  string identifier).
- Classes in main.ml2db.mulan.mulanarff used by main.ml2db.al.utility

  Class and Description

  IdInstance

  A Weka instance with identifier.

  IdMultiLabelInstances

  A set of identified Mulan Multi Label instances (each instance has a unique
  string identifier).
- Classes in main.ml2db.mulan.mulanarff used by main.ml2db.al.utility.confidence

  Class and Description

  IdInstance

  A Weka instance with identifier.

  IdMultiLabelInstances

  A set of identified Mulan Multi Label instances (each instance has a unique
  string identifier).
- Classes in main.ml2db.mulan.mulanarff used by main.ml2db.al.utility.globaldistance

  Class and Description

  IdInstance

  A Weka instance with identifier.

  IdMultiLabelInstances

  A set of identified Mulan Multi Label instances (each instance has a unique
  string identifier).
- Classes in main.ml2db.mulan.mulanarff used by main.ml2db.al.utility.hybrid

  Class and Description

  IdInstance

  A Weka instance with identifier.

  IdMultiLabelInstances

  A set of identified Mulan Multi Label instances (each instance has a unique
  string identifier).
- Classes in main.ml2db.mulan.mulanarff used by main.ml2db.al.utility.neighbours

  Class and Description

  IdInstance

  A Weka instance with identifier.

  IdMultiLabelInstances

  A set of identified Mulan Multi Label instances (each instance has a unique
  string identifier).
- Classes in main.ml2db.mulan.mulanarff used by main.ml2db.al.utility.pickers

  Class and Description

  IdInstance

  A Weka instance with identifier.

  IdMultiLabelInstances

  A set of identified Mulan Multi Label instances (each instance has a unique
  string identifier).
- Classes in main.ml2db.mulan.mulanarff used by main.ml2db.al.utility.pickers.byfrequency

  Class and Description

  IdInstance

  A Weka instance with identifier.

  IdMultiLabelInstances

  A set of identified Mulan Multi Label instances (each instance has a unique
  string identifier).
- Classes in main.ml2db.mulan.mulanarff used by main.ml2db.graphviz

  Class and Description

  IdInstance

  A Weka instance with identifier.

  IdMultiLabelInstances

  A set of identified Mulan Multi Label instances (each instance has a unique
  string identifier).
- Classes in main.ml2db.mulan.mulanarff used by main.ml2db.mulan.attributesfiltered

  Class and Description

  MulanArff

  Generates an Attribute Relation Format File (ARFF) in Mulan format (multiple
  class labels per instance).
- Classes in main.ml2db.mulan.mulanarff used by main.ml2db.mulan.learn.crosseval

  Class and Description

  IdMultiLabelInstances

  A set of identified Mulan Multi Label instances (each instance has a unique
  string identifier).
- Classes in main.ml2db.mulan.mulanarff used by main.ml2db.mulan.learn.traintest

  Class and Description

  IdMultiLabelInstances

  A set of identified Mulan Multi Label instances (each instance has a unique
  string identifier).
- Classes in main.ml2db.mulan.mulanarff used by main.ml2db.mulan.mulanarff

  Class and Description

  IdInstance

  A Weka instance with identifier.

  IdMultiLabelInstances

  A set of identified Mulan Multi Label instances (each instance has a unique
  string identifier).

  MulanArff

  Generates an Attribute Relation Format File (ARFF) in Mulan format (multiple
  class labels per instance).

  MulanAttributeFactory

  In the Mulan ARFF, the class attribute is not generated.

  MulanDataSetDbLoader

  Loads data from database to a Mulan data factory

  MulanDataSetGenerator

  Class

  MulanDataSetManager

  This class generates a data set (instances, attributes, classes) in Mulan
  style (for multi-label machine learning).

  NeighboursPrinter

  Class to store the neighbours of an instance and print them in a
  human/graphviz readable format.
- Classes in main.ml2db.mulan.mulanarff used by main.ml2db.mulan.mulanarff.database

  Class and Description

  IdMultiLabelInstances

  A set of identified Mulan Multi Label instances (each instance has a unique
  string identifier).
- Classes in main.ml2db.mulan.mulanarff used by main.ml2db.mulan.predict

  Class and Description

  IdInstance

  A Weka instance with identifier.

  IdMultiLabelInstances

  A set of identified Mulan Multi Label instances (each instance has a unique
  string identifier).
- Classes in main.ml2db.mulan.mulanarff used by main.ml2db.weka

  Class and Description

  IdMultiLabelInstances

  A set of identified Mulan Multi Label instances (each instance has a unique
  string identifier).
- Classes in main.ml2db.mulan.mulanarff used by test.ml2db.al

  Class and Description

  IdInstance

  A Weka instance with identifier.
- Classes in main.ml2db.mulan.mulanarff used by test.ml2db.al.utility.confidence

  Class and Description

  IdMultiLabelInstances

  A set of identified Mulan Multi Label instances (each instance has a unique
  string identifier).
- Classes in main.ml2db.mulan.mulanarff used by test.ml2db.al.utility.globaldistance

  Class and Description

  IdInstance

  A Weka instance with identifier.
- Classes in main.ml2db.mulan.mulanarff used by test.ml2db.mulan.mulanarff

  Class and Description

  IdMultiLabelInstances

  A set of identified Mulan Multi Label instances (each instance has a unique
  string identifier).

  MulanArff

  Generates an Attribute Relation Format File (ARFF) in Mulan format (multiple
  class labels per instance).

  MulanDataSetDbLoader

  Loads data from database to a Mulan data factory

  MulanDataSetGenerator

  Class

  MulanDataSetManager

  This class generates a data set (instances, attributes, classes) in Mulan
  style (for multi-label machine learning).

  MulanInstancesFiller

  Class

  Statistics

  Class exteded from Mulan
   Class for calculating statistics of a multilabel
  dataset

- Overview
- Package
- Class
- Use
- Tree
- Deprecated
- Index
- Help

- Prev
- Next

- Frames
- No Frames

- All Classes
